# Methimazole-Induced Goitrogenesis in an Adult Patient With the Syndrome of Resistance to Thyroid Hormone

**DOI:** 10.1177/2324709614555768

**Published:** 2014-10-14

**Authors:** Kathleen Glymph, Aidar R. Gosmanov

**Affiliations:** 1 University of Tennessee Health Science Center, Memphis, TN, USA

**Keywords:** resistance to thyroid hormone syndrome, methimazole, goiter

## Abstract

Patients with the syndrome of resistance to thyroid hormone (RTH) have clinical (tachycardia and anxiety) and biochemical (elevated thyroid hormones level) features of hyperthyroidism. Based on previous reports in pediatric patients with the RTH, antithyroid treatment in these patients is not indicated. Clinical and biochemical sequel of antithyroid therapy in an adult patient with RTH was not previously reported. A 63-year-old African American female with history of RTH was treated with a therapy consisting of methimazole 15 mg daily and atenolol. Methimazole treatment resulted in reduction in thyroid hormone level while the patient’s TSH increased with a peak of 24.88 mIU/L. Having achieved biochemical euthyroidism, the patient developed thyroid gland enlargement associated with progressive symptoms of dysphagia and dyspnea. Examination demonstrated globally enlarged firm thyroid gland with areas of nodularity in both lobes. A computed tomography of the neck showed enlarged thyroid gland with extension around bilateral sternocleidomastoid muscles and compression onto the trachea. Methimazole therapy was discontinued and patient was treated just on atenolol. Over 12 months following discontinuation of methimazole, the patient experienced marked clinical and radiographic improvement of the goiter size associated with TSH reduction to 1.26 mIU/L and modest free thyroxine increase as expected in RTH. It seems appealing to treat patients with the RTH with antithyroid medications. However, in these patients decrease in thyroid hormone levels will stimulate TSH production, which can, in turn, predispose to goiter formation. Our report supports prior observations in children with RTH that treatment with methimazole is not indicated in adult patients with RTH.

## Introduction

Resistance to thyroid hormone (RTH) is a rare syndrome characterized by decreased tissue sensitivity to thyroid hormone. Patients with this syndrome tend to have elevated thyroxine and/or elevated triiodothyronine levels with normal or elevated thyroid stimulating hormone (TSH).^1,2^ The clinical presentation is highly variable, ranging from isolated biochemical abnormalities to a mixture of hypo-thyroid and hyperthyroid symptoms and manifestations. Mutations in the thyroid hormone receptor β gene (THRB) have been identified as a cause.^1^ Resistance can occur at tissues that express the mutant THRB such as the hypo-thalamus, pituitary, and liver but not in tissues that express mainly the TRα such as the heart and cerebral cortex. Thus, RTH can manifest with symptoms of thyrotoxicosis, including tachycardia or irritability. Treatment of these syndromes has relied on the use of β-adrenergic blockers to control hyperadrenergic symptoms or levo-triiodothyronine to provide enough circulating thyroid hormone to reduce goiter size via suppression of TSH.^1^

The use of antithyroid medications in pediatric patients with RTH has been associated with goitrogenesis.^3^ From a pathophysiological standpoint, the use of methimazole or propylthiouracil in the patients with RTH may reduce the level of circulating thyroid hormones; however, it is unknown whether patients with RTH could derive any clinical benefits from the treatment with a thionomide. There is paucity of information on the clinical outcomes of thionomide use in adult patients with RTH.^3^ Here we report an adult patient with RTH who was treated with methima-zole but developed increase in TSH resulting goitrogenesis and compressive effects followed by the improvement of the symptoms after discontinuation of methimazole.

## Case Presentation

A 63-year-old African American female with history of RTH presented to our endocrinology clinic in April 2012 for a second opinion on the management of thyroid goiter. She complained of increased size of her goiter with symptoms of dysphagia, dyspnea while lying flat, tightness in her throat, and hoarse voice.

She initially presented to an outside endocrinology clinic in September of 2005 with symptoms of hyperthyroidism and elevated level of free thyroxine and was started on methimazole 15 mg daily and atenolol 50 mg twice daily. Despite decrease in thyroid hormone level during the methimazole therapy, her TSH remained unexpectedly normal or elevated, and therefore, other etiologies of hyper-thyroidism were considered. Following extensive work up, she was subsequently diagnosed with RTH with a defect in the THRB gene (R243W) in 2008. Despite this new diagnosis, she was continued on the aforementioned therapy that included methimazole. In late 2011 and early 2012, the patient noted increase in neck size and dysphagia, which prompted her to seek alternative explanations for thyroid gland size increase.

Her past medical history also included hypertension, gastroesophageal reflux disease, osteoarthritis, and prediabetes. During the evaluation in our office, her medi-cations included methimazole 15 mg daily, esomeprazole 40 mg daily, hydrochorothiazide 12.5 mg daily, echinacea 400 mg daily, and a multivitamin daily. She denied history of tobacco or alcohol use. On physical examination, her blood pressure was 129/79 mm Hg, and her heart rate was 64 beats/min. Body weight was 141 lb with a body mass index of 25.8 kg/m^2^. Her thyroid was markedly enlarged on both sides with areas of nodularity noted in bilateral lobes. The right lobe had marked lateral extension and the left lobe demonstrated submandibular growth. No bruits were auscultated. Her exam was otherwise unremarkable.

**Figure 1.**
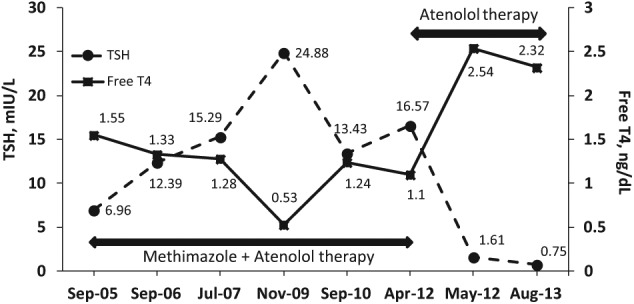
Time course of TSH and free T4 levels during methimazole therapy and after its discontinuation.

**Figure 2.**
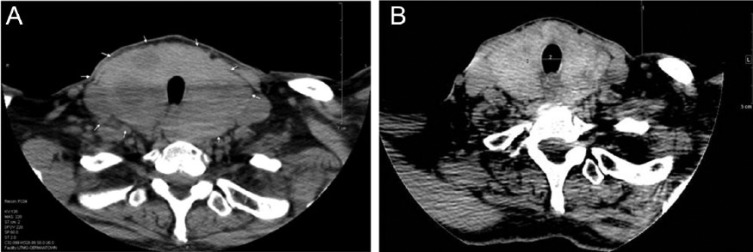
Thyroid gland size during methimazole therapy (A) and 1 year after methimazole discontinuation (B).

Given the recent onset of increase in thyroid gland size causing compressive symptoms, we reevaluated her previous laboratory data. On initial evaluation in 2005 and before initiation of methimazole therapy by an outside provider, laboratory studies revealed an elevated TSH of 6.96 (0.35-5.50 mIU/L), free T4 (FT4) of 1.55 (0.61-1.76 ng/dL; Figure 1), and total triiodothyronine (TT3) of 260 (85-205 ng/dL). Thyroid peroxidase antibodies were negative and thyroglobulin level was 129 (2-38 ng/mL) with negative thyroglobulin antibodies. When she was seen in our office in 2012 while continuing to receive methima-zole, her TSH was elevated at 16.57 mIU/L and FT4 was normal at 1.10 mIU/L. We ordered computed tomography (CT) of the neck that revealed a significantly enlarged thyroid with multiple bilateral nodules present with a moderate amount of tracheal compression (Figure 2A).

We believed that her increased goitrogenesis and associated symptoms were attributed to methimazole treatment with the latter causing persistent elevation of TSH in this patient with RTH. We stopped methimazole in April 2012, while continuing atenolol to control symptoms of hyperthyroidism and heart rate. The patient experienced steady improvement of her symptoms with subjective decline in the size of her goiter. Repeat CT of the neck done in August 2013 showed a notable decrease in the goiter size with no tracheal compression (Figure 2B). Follow-up laboratory showed a decrease and normalization of TSH associated with expected increase in FT4 of 2.31 mIU/L during the most recent clinical assessment (Figure 1).

## Discussion

Our patient with a history of documented RTH developed biochemical and clinical findings of TSH-induced goitrogenesis secondary to methimazole-induced thyroid hormone suppression. In these patients, it is imperative to understand the pathophysiology of RTH with specific focus on changes in TSH level when antithyroid therapy is being considered. TSH is known to be a thyroid cell growth factor that increases cell size and vascularity with overall thyroid enlargement and goiter development. Indeed, it has been reported that misdiagnosis of this condition has resulted in inappropriate therapy or management of one third of the cases with majority of the cases reported in pediatric population with RTH.^4^ This is the first report describing the detailed course of detrimental effects of the thionomide therapy in an adult patient with RTH.

RTH was first described in 1967 as a clinical syndrome associated with a defect in peripheral tissues’ response to thyroid hormones.^1,2^ The mutations found in RTH are inherited as autosomal dominant. There have been about 3000 cases reported, with approximately 85% of subjects identified as having heterozygous mutations in the THRβ gene, resulting in impaired T3 binding and/or function.^1,2^ These patients tend to exhibit signs of hyperthyroidism, such as tachycardia and anxiety. While peripheral tissues retain their sensitivity to thyroid hormones, higher concentrations of circulating thyroid hormones need to be present to provide a response at the level of the hypothalamus, pituitary, and liver in order to provide negative feedback in TSH release. The mechanism lies in the mutant THRβ interfering with the wild-type THRβ, known as a dominant-negative effect.^5^ This has been found to be the case in patients with the R243W THRB mutation, as was our patient’s diagnosis on genetic testing.^6^

## Conclusion

Although precise treatment for RTH is still being defined, understanding of pathophysiology of this condition is paramount to prevent iatrogenic adverse effects from inappropriate therapy. In our patient, the cessation of methimazole improved her symptoms and she continues to show signs of improvement in follow-up visits, remaining on atenolol as the only medication for her thyroid dysfunction. We believe that this case report lends several important lessons pertinent to the evaluation and management patients with hyperthyroi-dism. First, one should seek alternative explanations of hyperthyroidism etiology in patients who have elevated concentration of thyroid hormones and normal or mildly elevated TSH level. Second, initiation of antithyroid therapy in a patient with hyperthyroidism that results in further elevation of TSH should further suggest that pathophysiology of thyroid disease may differ from the course of most frequent causes of hyperthyroidism such as Graves’ disease or toxic goiter. Finally, the symptomatic treatment of hyperthyroidism with β-blockers should remain the favored therapy of RTH.
